# Onsager’s Non-Equilibrium Thermodynamics as Gradient Flow in Information Geometry

**DOI:** 10.3390/e27070710

**Published:** 2025-06-30

**Authors:** Tatsuaki Wada, Antonio Maria Scarfone

**Affiliations:** 1 Region of Electrical and Electronic Systems Engineering, Ibaraki University, Nakanarusawa-cho, Hitachi-shi 316-8511, Japan; 2 Istituto dei Sistemi Complessi, Consiglio Nazionale delle Ricerche (ISC-CNR), c/o Politecnico di Torino, Corso Duca degli Abruzzi, 24, 10129 Torino, Italy; antonio.scarfone@polito.it

**Keywords:** Onsager’s phenomenological equations, gradient flow, information geometry, irreversible thermodynamics

## Abstract

We consider Onsager’s non-equilibrium thermodynamics from the perspective of the gradient flow in information geometry. Assuming Onsager’s reciprocal relations, we can regard his phenomenological equations as gradient-flow equations and develop two different gradient-flow models. We consider their features and their relations. Both models are applied to the ideal gas and van der Waals gas.

## 1. Introduction

In a closed thermal system evolving from a non-equilibrium state that is not far from the equilibrium state, the thermodynamic entropic function S(X) can be expanded around the equilibrium state $X_{\mathrm{eq}}$ as

(1) $$\begin{array}{cc} S(X) & \approx S\left(X_{\mathrm{eq}}\right)+\frac{1}{2}{\left(\frac{\partial^{2}S\left(X\right)}{\partial X^{i}\partial X^{j}}\right)}_{X=X_{\mathrm{eq}}}\left(X^{i}-X_{\mathrm{eq}}^{i}\right)\left(X^{j}-X_{\mathrm{eq}}^{j}\right)\\ \; & =S\left(X_{\mathrm{eq}}\right)-\frac{1}{2}\;g_{ij}^{R}\left(X_{\mathrm{eq}}\right)\;\left(X^{i}-X_{\mathrm{eq}}^{i}\right)\left(X^{j}-X_{\mathrm{eq}}^{j}\right), \end{array}$$

where each $X_{i}$ is an extensive variable, and then, we introduce the Ruppeiner metric $g^{R}\left(X\right)$ [1]:

(2) $$\begin{array}{c} g_{ij}^{R}\left(X\right):=-\frac{\partial^{2}S\left(X\right)}{\partial X^{i}\partial X^{j}}. \end{array}$$

The components $g_{ij}^{R}\left(X\right)$ are positive definite and symmetric $g_{ij}^{R}\left(X\right)=g_{ji}^{R}\left(X\right)$, if the S(X) is a smooth concave function, i.e., −S(X) is convex. The entropy production rate can be described by

(3) $$\begin{array}{c} \frac{dS(X)}{dt}=\frac{\partial S(X)}{\partial X^{i}}\frac{dX^{i}}{dt}=a_{i}\;\frac{dX^{i}}{dt}, \end{array}$$

where

(4) $$\begin{array}{c} a_{i}:=\frac{\partial S(X)}{\partial X^{i}}, \end{array}$$

are the (conjugate) intensive variables, and $dX^{i}/dt$ are the thermodynamic fluxes of the variable $X^{i}$. Onsager [2,3] expands the fluxes as the linear combinations of the thermodynamic forces:

(5) $$\begin{array}{c} \frac{dX^{i}}{dt}=L^{ij}\;\frac{\partial S(X)}{\partial X^{i}}, \end{array}$$

which are known as Onsager’s phenomenological equations (OPE), the linear constitutive equations relating the fluxes and the forces with the condition that all $\partial S\left(X\right)/\partial X^{i}=0$ when all $dX^{i}/dt=0$. He assumes the coefficients $L^{ij}$ to be symmetric, i.e.,

(6) $$\begin{array}{c} L^{ij}=L^{ji}, \end{array}$$

whose symmetry is known as the reciprocal relations. Several studies have been performed concerning the OPE, e.g., Doi [4,5] extensively studied Onsager’s variational principle in soft matter.

Our previous studies on the gradient flow [6,7,8] in information geometry (IG) [9] showed some relations to different fields such as thermodynamics [10], geometric optics [10,11], analytical mechanics, and general relativity [12,13]. Among them, we pointed out [13] that Equation (5) can be regarded as the gradient-flow equation:

(7) $$\begin{array}{c} \frac{d\eta_{i}}{dt}=g_{ij}\left(\eta \right)\;\frac{\partial \left(-\Psi^{⋆}\left(\eta \right)\right)}{\partial \eta_{j}}, \end{array}$$

with respect to the η-potential function $\Psi^{⋆}\left(\eta \right)$ in IG, if we make the correspondence

(8) $$\begin{array}{c} X^{i}↔\eta_{i},\;L^{ij}↔g_{ij}\left(\eta \right),\;S\left(X\right)↔-\Psi^{⋆}\left(\eta \right),\;a_{i}\left(X\right):=\frac{\partial S(X)}{\partial X^{i}}↔-\theta^{i}=\frac{\partial \left(-\Psi^{⋆}\left(\eta \right)\right)}{\partial \eta_{i}}, \end{array}$$

where $g_{ij}\left(\eta \right)$ denotes the components of the Fisher matrix, and $\eta_{i}$ and $\theta^{i}$ are mutually dual affine coordinates in IG (see Appendix A for the details). In this correspondence, Onsager’s reciprocal relations (6) can be understood in IG as the symmetry of the metric $g_{ij}\left(\eta \right)=\partial \eta_{i}/\partial \theta^{j}=\partial \eta_{j}/\partial \theta^{i}=g_{ji}\left(\eta \right)$, which is due to integrability.

Research focusing on the mathematical scientific structures common to different fields is at the heart of SUURI engineering. SUURI is a Japanese word, which consists of the two Kanji characters: SUU (numbers, or mathematical things) and RI (reason, theory, or laws). SUURI is often translated as mathematics or applied physics, but they are not appropriate, and there is no direct counterpart in other languages. It treats natural and social phenomena as SUURI phenomena and deals with theorems and laws, theorizing and applying them. We explore this type of research on Onsager’s phenomenological equations from the perspective of the gradient-flow equations in IG.

The rest of the paper is organized as follows. In Section 2, we first consider the simple extension of OPE (5) based on our studies on the gradient-flow equations in IG. In this simple model, which we call the natural gradient-flow model, Onsager’s phenomenological coefficients are replaced with the Hessian of the negative thermodynamic entropic function −S(X). In Section 3, we adopt the components of the inverse of the metric *G* on a space spanned by the thermodynamic extensive variables $\left\{X^{i}\right\}$. We call this model the *G*-gradient-flow model. Section 4 applies these two models to the ideal gas and van der Waals gas. The final section is devoted to our conclusions. The basics of IG and the gradient flow in IG are summarized in Appendix A. Appendix B explains the connection to the cotangent bundle. This connection $\Gamma_{ij}\left(X,a\right)$ relates the flow $dX^{i}/dt$ in the tangent space TM to the flow $da_{i}/dt$ in the cotangent space $T^{⋆}M$. Appendix C shows the expression of the Levi–Civita connection of a metric *G* obtained from Jacobi matrix *J*. Throughout the paper, we use Einstein’s summation convention for a repeated index.

## 2. Natural Gradient-Flow Model

Here, we extend OPE (5) based on our studies [10,11,12,13] of the gradient-flow equations in IG. We apply the methods for deriving some important relations in IG to Onsager’s non-equilibrium thermodynamics. From the correspondence (8), as our first model, we introduce the coefficients $L_{ij}\left(X\right)$ as

(9) $$\begin{array}{c} L_{ij}\left(X\right):=-\frac{\partial a_{i}\left(X\right)}{\partial X^{j}}=-\frac{\partial^{2}S\left(X\right)}{\partial X^{i}\partial X^{j}}=g_{ij}^{R}\left(X\right). \end{array}$$

It is worth noting that the coefficients $L_{ij}\left(X\right)$ depend on *X* in general, whereas the coefficients $L_{ij}$ in OPE (5) are independent. One readily confirms the reciprocal relations as follows.

(10) $$\begin{array}{c} L_{ij}\left(X\right)=-\frac{\partial^{2}S\left(X\right)}{\partial X^{i}\partial X^{j}}=-\frac{\partial^{2}S\left(X\right)}{\partial X^{j}\partial X^{i}}=L_{ji}\left(X\right). \end{array}$$

Note that (9) means that the matrix L(X) is simultaneously a Jacobi matrix whose components are $-\partial a^{i}\left(X\right)/\partial X_{j}$ and a Hessian matrix of −S(X), similar to the distinct feature of the Fisher matrix *g* in IG (cf. (A4)).

We can introduce the total Legendre transform $S^{⋆}\left(a\right)$ of the thermodynamic entropic function S(X), i.e.,

(11) $$S^{⋆}\left(a\right)=X^{i}\left(a\right)\;a_{i}-S\left(X\left(a\right)\right),\;\frac{\partial S^{⋆}\left(a\right)}{\partial a_{i}}=X^{i}\left(a\right),\;\frac{\partial S(X)}{\partial X^{i}}=a_{i}\left(X\right).$$

Then, as the dual relation to (9), we have

(12) $$\begin{array}{c} L^{ij}\left(a\right)=-\frac{\partial X^{i}\left(a\right)}{\partial a_{j}}=-\frac{\partial^{2}S^{⋆}\left(a\right)}{\partial a_{i}\partial a_{j}}. \end{array}$$

Note that since the extensive variables $X^{i}$ and the intensive variables $a_{i}$ are related through (11), the coefficient $L^{ij}\left(a\right)$ can be regarded as a function of $X^{i}$, i.e., $L^{ij}\left(X\right):=L^{ij}\left(a\left(X\right)\right)$. With the coefficients $L^{ij}\left(X\right)$, as the gradient-flow equations corresponding to (A10) in IG, we propose

(13) $$\begin{array}{c} \frac{dX^{i}}{dt}=L^{ij}\left(X\right)\;\left(a_{j}-a_{j}^{\mathrm{eq}}\right)=L^{ij}\left(X\right)\left(\frac{\partial S(X)}{\partial X^{j}}-\frac{\partial S(X)}{\partial X^{j}}{|}_{X^{j}=X_{\mathrm{eq}}^{j}}\right), \end{array}$$

where $a-a^{\mathrm{eq}}$ denotes the thermodynamic force at a thermal state *X*. We assume that $a^{\mathrm{eq}}$ has a non-zero value in general, whereas in OPE (5), $a^{\mathrm{eq}}$ is assumed to be zero. Since in the fields of optimization and IG, the operator ${\overset{nat}{\nabla }}_{X^{i}}:=L^{ij}\left(X\right)\partial /\partial X^{j}$ is called the natural gradient [8,14], we call this model characterized by (13) the natural gradient-flow model.

Next, by using (9) and (13), we have

(14) $$\begin{array}{c} \frac{dX^{i}}{dt}=\underset{-L^{ij}\left(X\right)}{\underset{︸}{\frac{\partial X^{i}}{\partial a_{j}}}}\frac{da_{j}}{dt}=L^{ij}\left(X\right)\;\left(a_{j}-a_{j}^{\mathrm{eq}}\right). \end{array}$$

Consequently, it follows the linearized differential equations:

(15) $$\begin{array}{c} \frac{da_{i}}{dt}=-a_{i}+a_{i}^{\mathrm{eq}}, \end{array}$$

which are the dual equations to (13). Note that, whereas the original Equation (13) is nonlinear in general, the linearized Equation (15) is readily solved. As is clear from the above derivation process, the key point is that the coefficients $L^{ij}\left(X\right)$ are the components $-\partial X^{i}/\partial a_{j}$ of the Jacobi matrix.

By using (13), the entropy production rate (3) can be expressed as

(16) $$\begin{array}{c} \frac{dS(X)}{dt}=\underset{a_{i}}{\underset{︸}{\frac{\partial S(X)}{\partial X^{i}}}}\underset{L^{ij}\left(X\right)\left(a_{j}-a_{j}^{\mathrm{eq}}\right)}{\underset{︸}{\frac{dX^{i}}{dt}}}=a^{2}\left(X\right)-L^{ij}\left(X\right)a_{i}a_{j}^{\mathrm{eq}}, \end{array}$$

where we introduced the dissipation function:

(17) $$\begin{array}{c} a^{2}\left(X\right):=L^{ij}\left(X\right)\;\frac{\partial S(X)}{\partial X^{i}}\frac{\partial S(X)}{\partial X^{j}}=L^{ij}\left(X\right)\;a_{i}a_{j}={\left(g^{R}\left(X\right)\right)}^{ij}\;a_{i}a_{j}>0. \end{array}$$

It is convenient to introduce the function $\chi^{2}\left(X\right)$:

(18) $$\begin{array}{cc} \chi^{2}\left(X\right) & :=L^{ij}\left(X\right)\left(\frac{\partial S(X)}{\partial X^{i}}-\frac{\partial S(X)}{\partial X^{i}}{|}_{X^{i}=X_{\mathrm{eq}}^{i}}\right)\left(\frac{\partial S(X)}{\partial X^{j}}-\frac{\partial S(X)}{\partial X^{j}}{|}_{X^{j}=X_{\mathrm{eq}}^{j}}\right)\\ \; & =L^{ij}\left(X\right)\left(a_{i}\left(X\right)-a_{i}^{\mathrm{eq}}\left(X\right)\right)\left(a_{j}\left(X\right)-a_{j}^{\mathrm{eq}}\left(X\right)\right), \end{array}$$

which corresponds to (A13b) in IG.

The relation between the gradient flow in IG and Hamilton flow were pointed out in [6,7]. Boumuki and Noda [15] studied this relationship from the perspective of symplectic geometry. Chirco et al. [16] discussed Lagrangian and Hamiltonian dynamics in their non-parametric formalism. In our previous studies [11,13], we proposed the special type of Hamiltonian, which describes the gradient flow in IG. Based on the results, Equation (A12b) of the Hamilton flow in IG, we can construct the Hamiltonian:

(19) $$\begin{array}{c} H\left(X,a\right):=\frac{1}{2}L^{ij}\left(X\right)\;\left(a_{i}-a_{i}^{\mathrm{eq}}\right)\left(a_{j}-a_{j}^{\mathrm{eq}}\right)-\frac{1}{2}\chi^{2}\left(X\right), \end{array}$$

whose Hamilton flow is equivalent to the natural gradient flow (13) and its dual (15). Indeed, Hamilton’s equations of motion are

$$\{\begin{array}{l} \frac{dX^{i}}{dt}=\frac{\partial H}{\partial a_{i}}=L^{ij}(X)(a_{j}-a_{j}^{\mathrm{eq}}),\;\;\;\;\;\;\;\;\;\;\;\;\;\;\;\;\;\;\;\;\;\;\;\;\;\;\;\text{(20a)}\\ \frac{da_{i}}{dt}=-\frac{\partial H}{\partial X^{i}}=-\frac{1}{2}\frac{\partial L^{\mathrm{jk}}(X)}{\partial X^{i}}(a_{j}-a_{j}^{\mathrm{eq}})(a_{k}-a_{k}^{\mathrm{eq}})+\frac{1}{2}\frac{\partial \chi^{2}\left(X\right)}{\partial X^{i}}.\;\;\;\;\;\;\;\;\;\;\;\;\;\;\;\;\;\text{(20b)} \end{array}$$

The first equations are equivalent to (13). For the second equations, from (18), we have

(21) $$\begin{array}{cc} \frac{1}{2}\frac{\partial \chi^{2}\left(X\right)}{\partial X^{i}} & =\frac{1}{2}\frac{\partial L^{jk}\left(X\right)}{\partial X^{i}}\left(a_{j}-a_{j}^{\mathrm{eq}}\right)\left(a_{k}-a_{k}^{\mathrm{eq}}\right)+L^{jk}\left(X\right)\underset{-L_{ji}\left(X\right)}{\underset{︸}{\frac{\partial a_{j}}{\partial X^{i}}}}\left(a_{k}-a_{k}^{\mathrm{eq}}\right)\\ \; & =\frac{1}{2}\frac{\partial L^{jk}\left(X\right)}{\partial X^{i}}\left(a_{j}-a_{j}^{\mathrm{eq}}\right)\left(a_{k}-a_{k}^{\mathrm{eq}}\right)-\underset{\delta_{i}^{k}}{\underset{︸}{L^{jk}\left(X\right)L_{ji}\left(X\right)}}\left(a_{k}-a_{k}^{\mathrm{eq}}\right)\\ \; & =\frac{1}{2}\frac{\partial L^{jk}\left(X\right)}{\partial X^{i}}\left(a_{j}-a_{j}^{\mathrm{eq}}\right)\left(a_{k}-a_{k}^{\mathrm{eq}}\right)-a_{i}+a_{i}^{\mathrm{eq}}. \end{array}$$

Substituting this relation into the right-hand side in (20b), we obtain the linearized Equation (15).

One may wonder why the Hamiltonian (19) describes simultaneously the natural gradient-flow $dX^{i}/dt$ and its dual flow $da_{i}/dt$. In Appendix B, we explain the connection Γ(X,a) with the cotangent bundle. The connection Γ(X,a) relates the flow $dX^{i}/dt$ in the tangent space to the flow $da_{i}/dt$ in the cotangent space. The Hamiltonian (19) is constant along all horizontal curves and satisfies the distinctive relation (A40), from which the flow $dX^{i}/dt$ in tangent space and the flow $da_{i}/dt$ in cotangent space are related, as shown (A42), by the transport Equation (A17). Actually, the natural gradient flow Equation (13) and the dual linearized Equation (15) are related by

(22) $$\begin{array}{c} \frac{da_{i}}{dt}=-L_{ij}\left(X\right)\;\frac{dX^{i}}{dt}. \end{array}$$

## 3. G-Gradient-Flow Model

In the natural gradient-flow model, the coefficients $L_{ij}\left(X\right)$ are important ingredients and equivalent to the Hessian of −S(X), and also to the Ruppeiner metric [1]. However, in order to obtain the coefficients $L_{ij}\left(X\right)$ in (9), it requires an expression of the thermodynamic entropic function S(X) as a function of the extensive variables $X^{i}$. It is difficult to determine an explicit expression of the thermodynamic entropy, in general. Thermodynamic systems are often characterized by a set of equations of thermodynamic state, which are experimentally determined. Vaz [17] provided the method for obtaining the metric *G* in the space spanned by the extensive variables $\left\{X^{i}\right\}$ from a set of equations of thermodynamic state. Here, we propose another model (*G*-gradient-flow equations) based on Vaz’s method.

Let us consider the coordinate transformation from $X=\{X^{i}\}$ to a function $\phi^{b}\left(X\right)$ of *X*,

(23) $$\begin{array}{c} \frac{\partial }{\partial \phi^{b}}=\frac{\partial X^{i}}{\partial \phi^{b}}\;\frac{\partial }{\partial X^{i}}=J_{b}^{i}\left(X\right)\;\frac{\partial }{\partial X^{i}},\;i,b=1,2,\ldots ,n, \end{array}$$

where ${J_{b}}^{i}\left(X\right):=\partial X^{i}/\partial \phi^{b}$ are the components of the Jacobi matrix *J*. The meaning of each function $\phi^{b}\left(X\right)$ is shown in (30). Similarly, the inverse relations are given by

(24) $$\begin{array}{c} d\phi^{b}=\frac{\partial \phi^{b}}{\partial X^{i}}\;dX^{i}={J_{i}}^{b}\left(X\right)\;dX^{i},\;i,b=1,2,\ldots ,n. \end{array}$$

The following relations are satisfied.

(25) $$\begin{array}{c} {J_{b}}^{i}\left(X\right)\;{J_{i}}^{c}\left(X\right)=\frac{\partial X^{i}}{\partial \phi^{b}}\;\frac{\partial \phi^{c}}{\partial X^{i}}=\frac{\partial \phi^{c}}{\partial \phi^{b}}=\delta_{b}^{c},\;{J_{i}}^{b}\left(X\right)\;{J_{b}}^{j}\left(X\right)=\frac{\partial \phi^{b}}{\partial X^{i}}\;\frac{\partial X^{j}}{\partial \phi^{b}}=\frac{\partial X^{j}}{\partial X^{i}}=\delta_{i}^{j}. \end{array}$$

Thus, (23) describes the transformation rule from the frame consisting of the coordinate basis $\left\{\partial /\partial X^{i}\right\}$ to the frame consisting of the basis $\left\{\partial /\partial \phi^{b}\right\}$. Their dual bases are $\left\{dX^{i}\right\}$ and $\left\{d\phi^{b}\right\}$, respectively, and they satisfy

(26) $$\begin{array}{c} dX^{i}\frac{\partial }{\partial X^{j}}=\delta_{j}^{i},\;d\phi^{b}\frac{\partial }{\partial \phi^{c}}=\delta_{c}^{b}. \end{array}$$

In general, equilibrium thermodynamic systems with *n*-independent macroscopic variables are completely described by the *n*-independent equations of state, which, in some cases, can be cast into the following form [10,17],

(27) $$\begin{array}{c} {J_{b}}^{i}\left(X\right)\;a_{i}=E_{b},\;i,b=1,2,\ldots n, \end{array}$$

where $a_{i}$ is given in (4), and each $E_{b}$ is an independent constant. We can assign $\left\{E_{b}\right\}$ as the components in an orthogonal basis $\left\{\partial /\partial \phi^{b}\right\}$ with the invertible constant diagonal matrix η. In other words, the frame consisting of the orthogonal basis $\left\{\partial /\partial \phi^{b}\right\}$ is Cartan’s moving frame. In general, the frame $\left\{\partial /\partial X^{i}\right\}$ is non-orthogonal and is characterized by a metric tensor *G*, whose components $G_{ij}\left(X\right)$ are related by

(28) $$\begin{array}{c} G_{ij}\left(X\right)={\eta_{bc}\;J_{i}}^{b}\left(X\right){J_{j}}^{c}\left(X\right). \end{array}$$

The Jacobi matrix *J* relates the non-orthogonal frame $\left\{\partial /\partial X^{i}\right\}$ with the local orthogonal frame $\left\{\partial /\partial \phi^{b}\right\}$ as shown in (27).

Next, by inverting (27), we have

(29) $$\begin{array}{c} \frac{\partial S(X)}{\partial X^{i}}=a_{i}=E_{b}\;{J_{i}}^{b}\left(X\right)=\frac{\partial (E_{b}\;\phi^{b}\left(X\right))}{\partial X^{i}}, \end{array}$$

which implies that the entropic function is expressed in the form:

(30) $$\begin{array}{c} S\left(X\right)=E_{b}\;\phi^{b}\left(X\right), \end{array}$$

except for the constant of integration. In this way, when the thermodynamic equations of state are cast into the form (27), the thermodynamic entropic function S(X) is decomposed in the form (30), which consists of the sum of the product of a constant $E_{b}$ and a function $\phi^{b}\left(X\right)$.

The inner product in the orthogonal frame $\left\{\partial /\partial \phi^{b}\right\}$ with a diagonal metric tensor η and that in the non-orthogonal frame $\left\{\partial /\partial X^{i}\right\}$ with a metric tensor *G* are related by

(31) $$\begin{array}{c} G^{ij}\left(X\right)\;a_{i}a_{j}=\eta^{bc}\;E_{b}E_{c}. \end{array}$$

We can regard this relation as the generalized eikonal equation [10] (or Hamilton–Jacobi equation):

(32) $$\begin{array}{c} G^{ij}\left(X\right)\;\frac{\partial S(X)}{\partial X^{i}}\frac{\partial S(X)}{\partial X^{j}}=E^{2}, \end{array}$$

where

(33) $$\begin{array}{c} E^{2}:=\eta^{bc}E_{b}E_{c}, \end{array}$$

is a positive constant. From Equations (27) and (31), it follows that

(34) $$\begin{array}{c} G^{ij}\left(X\right)=\eta^{bc}\;{J_{b}}^{i}\left(X\right){J_{c}}^{j}\left(X\right). \end{array}$$

It is worth noting that a Jacobi matrix J(X) is determined by $n^{2}$ components, whereas a Riemann metric G(X) has n(n+1)/2 components. Consequently, the metric G(X) is obtained from a given Jacobi matrix J(X) as (28) and (34), whereas the converse is not possible in general, since $n\left(n+1\right)/2<n^{2}$ for n>1.

Now, we introduce the *G*-gradient-flow model as follows.

(35) $$\begin{array}{c} \frac{dX^{i}}{dt}=G^{ij}\left(X\right)\;\left(a_{j}-a_{j}^{\mathrm{eq}}\right)=G^{ij}\left(X\right)\;\left(\frac{\partial S(X)}{\partial X^{i}}-\frac{\partial S(X)}{\partial X^{j}}{|}_{X^{j}=X_{\mathrm{eq}}^{j}}\right). \end{array}$$

Note that, since η is a diagonal matrix, G(X) is symmetric under exchanging of indices, i.e, the reciprocal relations are satisfied. Since the coefficient $G_{ij}\left(X\right)$ comprises the components of the metric G(X), we call this model the *G*-gradient-flow model.

The entropic rate in this model is

(36) $$\begin{array}{cc} \frac{dS(X)}{dt} & =\underset{a_{i}}{\underset{︸}{\frac{\partial S(X)}{\partial X^{i}}}}\underset{G^{ij}\left(X\right)\left(a_{j}-a_{j}^{\mathrm{eq}}\right)}{\underset{︸}{\frac{dX^{i}}{dt}}}=G^{ij}\left(X\right)\underset{{J_{i}}^{b}\left(X\right)E_{b}}{\underset{︸}{a_{i}}}\left(\underset{{J_{j}}^{c}\left(X\right)E_{c}}{\underset{︸}{a_{j}}}-\underset{{J_{j}}^{c}\left(X_{\mathrm{eq}}\right)E_{c}}{\underset{︸}{a_{j}^{\mathrm{eq}}}}\right)\\ \; & =\underset{\eta^{bc}}{\underset{︸}{G^{ij}\left(X\right){J_{i}}^{b}\left(X\right){J_{j}}^{c}\left(X\right)}}E_{b}E_{c}-G^{ij}\left(X\right){J_{i}}^{b}\left(X\right){J_{j}}^{c}\left(X_{\mathrm{eq}}\right)E_{b}E_{c}\\ \; & =\eta^{bc}E_{b}E_{c}-G^{ij}\left(X\right){J_{i}}^{b}\left(X\right){J_{j}}^{c}\left(X_{\mathrm{eq}}\right)E_{b}E_{c}=E^{2}-G^{ij}\left(X\right){J_{i}}^{b}\left(X\right){J_{j}}^{c}\left(X_{\mathrm{eq}}\right)E_{b}E_{c}. \end{array}$$

From (29), we obtain the dual equations with respect to (35) as follows.

(37) $$\begin{array}{cc} \frac{da_{i}}{dt} & =E_{b}\;\frac{d{J_{i}}^{b}\left(X\right)}{dt}=E_{b}\;\frac{\partial }{\partial X^{j}}\underset{\frac{\partial \phi^{b}\left(X\right)}{\partial X^{i}}}{\underset{︸}{{J_{i}}^{b}\left(X\right)}}\underset{G^{jk}\left(X\right)\left(a_{k}-a_{k}^{\mathrm{eq}}\right)}{\underset{︸}{\frac{dX^{j}}{dt}}}\\ \; & =\underset{-L_{ij}\left(X\right)}{\underset{︸}{\frac{\partial^{2}S\left(X\right)}{\partial X^{i}\partial X^{j}}}}G^{jk}\left(X\right)\left(a_{k}-a_{k}^{\mathrm{eq}}\right)=-L_{ij}\left(X\right)G^{jk}\left(X\right)\left(a_{k}-a_{k}^{\mathrm{eq}}\right). \end{array}$$

Combining (35) and (37), we have

(38) $$\begin{array}{c} \frac{da_{i}}{dt}=-L_{ij}\left(X\right)\;\frac{dX^{j}}{dt}, \end{array}$$

which are the transport equations in (A17). As explained in Appendix B, the connection $\Gamma_{ij}\left(X,a\right)$ to the cotangent bundle relates the flow $dX^{i}/dt$ in the tangent space TM to the flow $da_{i}/dt$ in the cotangent space $T^{⋆}M$. Hence, we find that −L(X(a)) plays a role of the connection Γ(X,a) to a cotangent bundle.

This relation (Γ(X,a)=−L(X)) can be confirmed as follows. As shown in Appendix C, the coefficients ΓkGij(X) of the Levi–Civita connection with respect to *G* are obtained in (A47), i.e.,

(39) ΓkGij(X)=Jkb(X)∂Jjb(X)∂Xi.

Then, the associated linear connection Γ(X,a) in (A25) becomes

(40) Γ(X,a)=ΓkGij(X)ak=Jkb(X)ak∂Jjb(X)∂Xi=∂Xk∂ϕb(X)∂S(X)∂Xk∂2ϕb(X)∂Xi∂Xj=∂S(X)∂ϕb︸Eb∂2ϕb(X)∂Xi∂Xj=∂2∂Xi∂XjEbϕb(X)︸S(X)=∂2S(X)∂Xi∂Xj=−Lij(X).

## 4. Applications

Here, we apply the natural- and *G*-gradient-flow models to ideal gas and van der Waals gas.

### 4.1. Ideal Gas

Ideal gas is a simple model for a dilute gas, which is characterized by the following equations of state:

(41) $$\begin{array}{c} u=C_{v}T,\;P\;v=RT, \end{array}$$

where *u* denotes the molar internal energy, *v* the molar volume, *T* the absolute temperature, *P* the pressure, *R* the gas constant, and $C_{v}$ the molar heat capacity at constant volume.

Now, the extensive variables are X=(u,v), and Equation (41) can be cast into the form (27):

(42) $$\begin{array}{c} \underset{{J_{b}}^{i}\left(X\right)}{\underset{︸}{\left(\begin{array}{cc} u & 0\\ 0 & v \end{array}\right)}}\underset{a_{i}}{\underset{︸}{\left(\begin{array}{c} \frac{1}{T}\\ \frac{P}{T} \end{array}\right)}}=\underset{E_{b}}{\underset{︸}{\left(\begin{array}{c} C_{v}\\ R \end{array}\right),}} \end{array}$$

where

(43) $$\begin{array}{c} {J_{b}}^{i}\left(X\right)=\left(\begin{array}{cc} u & 0\\ 0 & v \end{array}\right),\;\;a_{i}=\left(\begin{array}{c} \frac{1}{T}\\ \frac{P}{T} \end{array}\right),\;E_{b}=\left(\begin{array}{c} C_{v}\\ R \end{array}\right), \end{array}$$

where we set the conserved quantity $E_{u}=C_{v}$ and $E_{v}=R$. From the definition of ${J_{i}}^{b}\left(X\right):=\partial \phi^{b}/\partial X^{i}$, the components ${J_{i}}^{b}\left(X\right)$ are related by

(44) $$\begin{array}{c} {J_{i}}^{b}\left(X\right)=\left(\begin{array}{cc} \frac{1}{u} & 0\\ 0 & \frac{1}{v} \end{array}\right)=\left(\begin{array}{cc} \frac{\partial }{\partial u}\phi^{u}\left(X\right) & \frac{\partial }{\partial u}\phi^{v}\left(X\right)\\ \frac{\partial }{\partial v}\phi^{u}\left(X\right) & \frac{\partial }{\partial v}\phi^{v}\left(X\right) \end{array}\right), \end{array}$$

from which we obtain

(45) $$\begin{array}{c} \phi^{u}\left(X\right)=\ln u,\;\phi^{v}\left(X\right)=\ln v. \end{array}$$

Then, from (30), the thermodynamic entropic function of the ideal gas model is expressed as

(46) $$\begin{array}{c} S\left(X\right)=C_{v}\ln u+R\ln v. \end{array}$$

Setting $\eta =\mathrm{diag}(C_{v},R)$ and $\eta^{-1}=\mathrm{diag}\left(1/C_{v},1/R\right)$, we have

(47) $$\begin{array}{c} G\left(X\right)=\left(\begin{array}{cc} \frac{C_{v}}{u^{2}} & 0\\ 0 & \frac{R}{v^{2}} \end{array}\right), \end{array}$$

and

(48) $$\begin{array}{c} G^{-1}\left(X\right)=\left(\begin{array}{cc} \frac{u^{2}}{C_{v}} & 0\\ 0 & \frac{v^{2}}{R} \end{array}\right). \end{array}$$

The matrix L(X) of the ideal gas model is

(49) $$\begin{array}{c} L\left(X\right)=\left(\begin{array}{cc} \frac{C_{v}}{u^{2}} & 0\\ 0 & \frac{R}{v^{2}} \end{array}\right), \end{array}$$

which is same as the metric G(X) in (47). Hence, there is no difference between the natural gradient-flow equations and *G*-gradient-flow equations for the ideal gas model. This is because this model is too simple. In contrast, as we show in the next subsection for van der Waals gas that both gradient-flow equations are different.

Now, we consider the gradient-flow Equation (13) for the ideal gas model.

(50) $$\begin{array}{c} \frac{du}{dt}=u-\frac{u^{2}}{u_{\mathrm{eq}}},\;\frac{dv}{dt}=v-\frac{v^{2}}{v_{\mathrm{eq}}}. \end{array}$$

Instead of solving these nonlinear differential equations, it is easier to solve their dual linear Equation (15):

(51) $$\begin{array}{c} \frac{d}{dt}\left(\frac{1}{T}\right)=-\frac{1}{T}+\frac{1}{T_{\mathrm{eq}}},\;\frac{d}{dt}\left(\frac{P}{T}\right)=-\frac{P}{T}+\frac{P_{\mathrm{eq}}}{T_{\mathrm{eq}}}, \end{array}$$

where $T_{\mathrm{eq}}$ and $P_{\mathrm{eq}}$ are the equilibrium temperature and pressure, respectively. The solutions are

(52) $$\begin{array}{c} \frac{1}{T(t)}=\left(\frac{1}{T_{0}}-\frac{1}{T_{\mathrm{eq}}}\right)\exp\left(-t\right)+\frac{1}{T_{\mathrm{eq}}},\;P\left(t\right)=\frac{\left(P_{0}-P_{\mathrm{eq}}\right)\exp\left(-t\right)}{\left(1-\frac{T_{0}}{T_{\mathrm{eq}}}\right)\exp\left(-t\right)+\frac{T_{0}}{T_{\mathrm{eq}}}}+P_{\mathrm{eq}}, \end{array}$$

where $T_{0}$ and $P_{0}$ are the initial temperature and pressure, respectively. From the explicit expression of the entropic function $S_{\mathrm{ig}}\left(X\right)$ in (46), we obtain

(53) $$\begin{array}{c} a_{u}=\frac{1}{T}=\frac{\partial S}{\partial u}=\frac{C_{v}}{u},\;a_{v}=\frac{P}{T}=\frac{\partial S}{\partial v}=\frac{R}{v}=\frac{C_{v}}{u}\left(\frac{R\;u}{C_{v}\;v}\right). \end{array}$$

From these relations, we obtain $P=Ru/(C_{v}v)$. The relations between the extensive variables X=(u,v) and the intensive variables a=(1/T,P/T) are simple and separated. Consequently, we can readily obtain the solutions of the gradient-flow Equation (50) as

(54) $$\begin{array}{c} u\left(t\right)=C_{v}T\left(t\right)=\frac{u_{\mathrm{eq}}}{1+\left(\frac{u_{\mathrm{eq}}}{u_{0}}-1\right)\exp\left(-t\right)},\;v\left(t\right)=R\frac{P(t)}{T(t)}=\frac{v_{\mathrm{eq}}}{1+\left(\frac{v_{\mathrm{eq}}}{v_{0}}-1\right)\exp\left(-t\right)}. \end{array}$$

The entropic production rate is obtained by either (16) or (36). Since the two models are same for the ideal gas, we obtain $E^{2}=a^{2}=C_{v}+R=C_{p}$ (by Mayer’s relation) and

$$\begin{array}{c} G^{ij}\left(X\right){J_{i}}^{b}\left(X\right){J_{j}}^{c}\left(X_{\mathrm{eq}}\right)E_{b}E_{c}=L^{ij}\left(X\right)a_{i}\left(X\right)a_{j}\left(X_{\mathrm{eq}}\right)=C_{v}\frac{u(t)}{u_{\mathrm{eq}}}+R\frac{v(t)}{v_{\mathrm{eq}}}. \end{array}$$

Then, we obtain

$$\begin{array}{c} \frac{dS(t)}{dt}=C_{v}\left(1-\frac{u(t)}{u_{\mathrm{eq}}}\right)+R\left(1-\frac{v(t)}{v_{\mathrm{eq}}}\right). \end{array}$$

### 4.2. van der Waals Gas

Here, we consider the natural- and *G*-gradient-flow of the van der Waals gas model, which is characterized by the following equations of state:

(55) $$\begin{array}{c} u+\frac{a}{v}=C_{v}T,\;\left(P+\frac{a}{v^{2}}\right)\left(v-b\right)=RT, \end{array}$$

where a and b are the real parameters. The first equation of (55) states the equipartition theorem, and the second equation states the equation of state by van der Waals [18]. The term $a/v^{2}$ accounts for the long-range attractive forces that increase the pressure, and the b term accounts for the short-range repulsive forces that decrease the volume available to molecules. In the limit of a=b=0, the van der Waals gas model reduces to the ideal gas model.

Now, we have the extensive variables X=(u,v), and the equations in (55) are cast into the form (27):

(56) $$\begin{array}{c} \underset{{J_{b}}^{i}\left(X\right)}{\underset{︸}{\left(\begin{array}{cc} u+\frac{a}{v} & 0\\ \frac{a}{v^{2}}\left(v-b\right) & \;\;v-b \end{array}\right)}}\underset{a_{i}}{\underset{︸}{\left(\begin{array}{c} \frac{1}{T}\\ \frac{P}{T} \end{array}\right)}}=\underset{E_{b}}{\underset{︸}{\left(\begin{array}{c} C_{v}\\ R \end{array}\right),}} \end{array}$$

where

(57) $$\begin{array}{c} {J_{b}}^{i}\left(X\right)=\left(\begin{array}{cc} u+\frac{a}{v} & 0\\ \frac{a}{v^{2}}\left(v-b\right) & \;\;v-b \end{array}\right),\;\;a_{i}=\left(\begin{array}{c} \frac{1}{T}\\ \frac{P}{T} \end{array}\right),\;E_{b}=\left(\begin{array}{c} C_{v}\\ R \end{array}\right). \end{array}$$

The components ${J_{i}}^{b}\left(X\right)$ are

(58) $$\begin{array}{c} {J_{i}}^{b}\left(X\right)=\left(\begin{array}{cc} \frac{1}{u+\frac{a}{v}} & 0\\ -\frac{a}{v^{2}\left(u+\frac{a}{v}\right)} & \;\;\frac{1}{v-b} \end{array}\right)=\left(\begin{array}{cc} \frac{\partial }{\partial u}\phi^{u}\left(X\right) & \frac{\partial }{\partial u}\phi^{v}\left(X\right)\\ \frac{\partial }{\partial v}\phi^{u}\left(X\right) & \frac{\partial }{\partial v}\phi^{v}\left(X\right) \end{array}\right), \end{array}$$

where

(59) $$\begin{array}{c} \phi^{u}\left(X\right)=\ln\left(u+\frac{a}{v}\right),\;\phi^{v}\left(X\right)=\ln\left(v-b\right). \end{array}$$

The thermodynamic entropic function S(X) can be obtained as

(60) $$\begin{array}{c} S\left(X\right)=C_{v}\ln\left(u+\frac{a}{v}\right)+R\ln\left(v-b\right). \end{array}$$

Setting $\eta =\mathrm{diag}(C_{v},R)$ and $\eta^{-1}=\mathrm{diag}\left(1/C_{v},1/R\right)$, we have

(61) $$\begin{array}{c} G\left(X\right)=\left(\begin{array}{cc} \frac{C_{v}}{{\left(u+\frac{a}{v}\right)}^{2}} & -\frac{C_{v}\frac{a}{v^{2}}}{{\left(u+\frac{a}{v}\right)}^{2}}\\ -\frac{C_{v}\frac{a}{v^{2}}}{{\left(u+\frac{a}{v}\right)}^{2}} & \frac{C_{v}\frac{a^{2}}{v^{4}}}{{\left(u+\frac{a}{v}\right)}^{2}}+\frac{R}{{\left(v-b\right)}^{2}} \end{array}\right), \end{array}$$

and

(62) $$\begin{array}{c} G^{-1}\left(X\right)=\left(\begin{array}{cc} \frac{{\left(u+\frac{a}{v}\right)}^{2}}{C_{v}}+\frac{\frac{a^{2}}{v^{4}}{\left(v-b\right)}^{2}}{R} & \frac{\frac{a}{v^{2}}{\left(v-b\right)}^{2}}{R}\\ \frac{\frac{a}{v^{2}}{\left(v-b\right)}^{2}}{R} & \;\frac{{\left(v-b\right)}^{2}}{R} \end{array}\right). \end{array}$$

The non-zero elements of the Levi–Civita connection are

(63) ΓuGuu(X)=−1u+av,ΓuGuv(X)=ΓuGvu(X)=av2u+av,ΓuGvv(X)=av2−2abv3v−b−a2v4u+av,ΓvGvv(X)=−1v−b.

The straightforward calculations lead to all components ${R^{\ell }}_{ijk}\left(X\right)$ of Riemann curvature tensor being zero; hence, the scalar curvature of the *G*-gradient-flow model is zero.

The matrix L(X) is

(64) $$\begin{array}{c} L\left(X\right)=\left(\begin{array}{cc} \frac{C_{v}}{{\left(u+\frac{a}{v}\right)}^{2}} & -\frac{aC_{v}}{v^{2}{\left(u+\frac{a}{v}\right)}^{2}}\\ -\frac{aC_{v}}{v^{2}{\left(u+\frac{a}{v}\right)}^{2}} & \;-\frac{aC_{v}\left(a+2uv\right)}{v^{4}{\left(u+\frac{a}{v}\right)}^{2}}+\frac{R}{{\left(v-b\right)}^{2}} \end{array}\right). \end{array}$$

(65) $$\begin{array}{c} L^{-1}\left(X\right)=\frac{\left(u+\frac{a}{v}\right){\left(v-b\right)}^{2}}{R\left(u+\frac{a}{v}\right)-2C_{v}\frac{a}{v^{3}}{\left(v-b\right)}^{2}}\left(\begin{array}{cc} -\frac{a(a+2uv)}{v^{4}}+\frac{R{\left(u+\frac{a}{v}\right)}^{2}}{C_{v}{\left(v-b\right)}^{2}} & \frac{a}{v^{2}}\\ \frac{a}{v^{2}} & 1 \end{array}\right). \end{array}$$

Then, the natural gradient-flow Equation (13) is

(66) $$\begin{array}{c} \left(\begin{array}{c} \frac{du}{dt}\\ \frac{dv}{dt} \end{array}\right)=L^{-1}\left(X\right)\;\left(\begin{array}{c} \frac{C_{v}}{u+\frac{a}{v}}-\frac{C_{v}}{u_{\mathrm{eq}}+\frac{a}{v_{\mathrm{eq}}}}\\ \frac{R}{\left(v-b\right)}-\frac{C_{v}a}{v^{2}\left(u+\frac{a}{v}\right)}-\frac{R}{\left(v_{\mathrm{eq}}-b\right)}+\frac{C_{v}a}{v_{\mathrm{eq}}^{2}\left(u_{\mathrm{eq}}+\frac{a}{v_{\mathrm{eq}}}\right)} \end{array}\right). \end{array}$$

The dual linear Equation (15) for the van der Waals gas model is the same as that (51) for the ideal gas model. From the explicit expression (60) of the entropic function, we obtain

(67) $$\begin{array}{c} a_{u}=\frac{1}{T}=\frac{C_{v}}{u+\frac{a}{v}},\;a_{v}=\frac{P}{T}=\frac{R}{v-b}-\frac{C_{v}\frac{a}{v^{2}}}{u+\frac{a}{v}}=\frac{C_{v}}{u+\frac{a}{v}}\left(\frac{R\left(u+\frac{a}{v}\right)}{C_{v}\left(v-b\right)}-\frac{a}{v^{2}}\right), \end{array}$$

from which we obtain

(68) $$\begin{array}{c} P\left(t\right)=\frac{R\left(u+\frac{a}{v}\right)}{C_{v}\left(v-b\right)}-\frac{a}{v^{2}}. \end{array}$$

Next, we consider the *G*-gradient-flow model. From (35) and (66), after straightforward but tedious calculations, we have

(69a) $$\begin{array}{cc} \frac{du}{dt} & =u+\frac{a}{v}+\frac{a}{v^{2}}\left(v-b\right)-\frac{{\left(u+\frac{a}{v}\right)}^{2}}{u_{\mathrm{eq}}+\frac{a}{v_{\mathrm{eq}}}}-\frac{a}{v^{2}}\frac{{\left(v-b\right)}^{2}}{v_{\mathrm{eq}}-b}-\frac{C_{v}\frac{a^{2}}{v^{2}}{\left(v-b\right)}^{2}}{R\left(u_{\mathrm{eq}}+\frac{a}{v_{\mathrm{eq}}}\right)}\left(\frac{1}{v^{2}}-\frac{1}{v_{\mathrm{eq}}^{2}}\right), \end{array}$$

(69b) $$\begin{array}{cc} \frac{dv}{dt} & =v-b-\frac{{\left(v-b\right)}^{2}}{v_{\mathrm{eq}}-b}-\frac{C_{v}a{\left(v-b\right)}^{2}}{R\left(u_{\mathrm{eq}}+\frac{a}{v_{\mathrm{eq}}}\right)}\left(\frac{1}{v^{2}}-\frac{1}{v_{\mathrm{eq}}^{2}}\right). \end{array}$$

The dual Equation (37) for the van der Waals gas model is obtained by using

(70) $$\begin{array}{c} L_{ij}\left(X\right)G^{jk}\left(X\right)=\left(\begin{array}{cc} 1 & 0,\\ -\frac{2C_{v}a^{2}{\left(v-b\right)}^{2}}{Rv^{5}\left(u+\frac{a}{v}\right)} & 1-\frac{2C_{v}a{\left(v-b\right)}^{2}}{Rv^{3}\left(u+\frac{a}{v}\right)} \end{array}\right), \end{array}$$

so that

(71a) $$\begin{array}{cc} \frac{d}{dt}\left(\frac{1}{T}\right) & =-\frac{1}{T}+\frac{1}{T_{\mathrm{eq}}}, \end{array}$$

(71b) $$\begin{array}{cc} \frac{d}{dt}\left(\frac{P}{T}\right) & =-\frac{2C_{v}a^{2}{\left(v-b\right)}^{2}}{Rv^{5}\left(u+\frac{a}{v}\right)}\left(\frac{1}{T}-\frac{1}{T_{\mathrm{eq}}}\right)-\left(1-\frac{2C_{v}a{\left(v-b\right)}^{2}}{Rv^{3}\left(u+\frac{a}{v}\right)}\right)\left(\frac{P}{T}-\frac{P_{\mathrm{eq}}}{T_{\mathrm{eq}}}\right). \end{array}$$

In contrast to the case of the natural gradient-flow model, the second differential equation is nonlinear.

**Figure 1:**
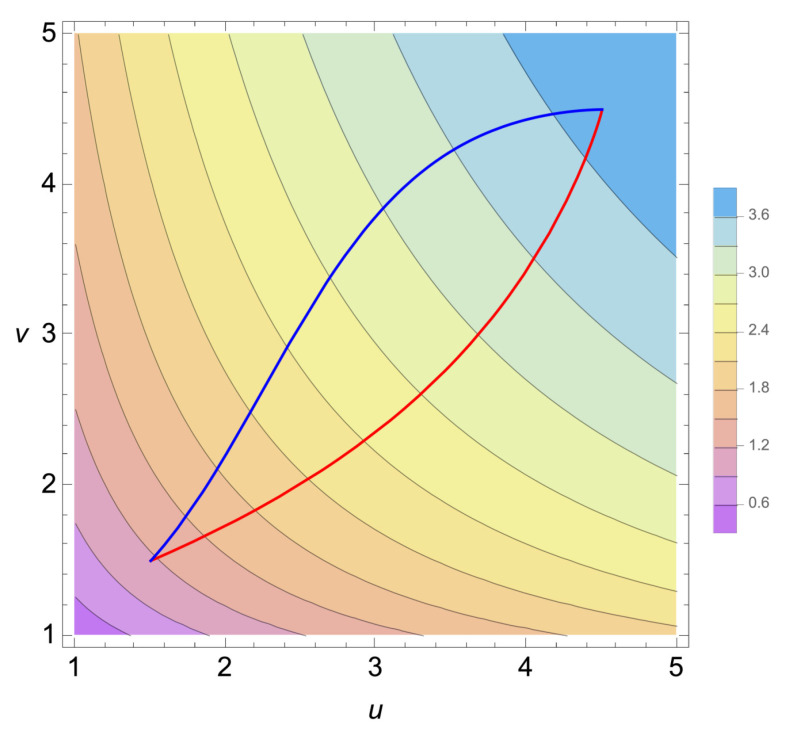
The contour plot of the entropic function (60). The trajectories (u(t), v(t)) of the natural gradient flow (blue curve) and *G*-gradient flow (red curve) are also plotted. We set $C_{v}=3/2$ (a monoatomic gas), $R=1,\;a=1,\;b=0.5,\;u\left(0\right)=v\left(0\right)=1.5,\;u_{\mathrm{eq}}=v_{\mathrm{eq}}=4.5$.

**Figure 2:**
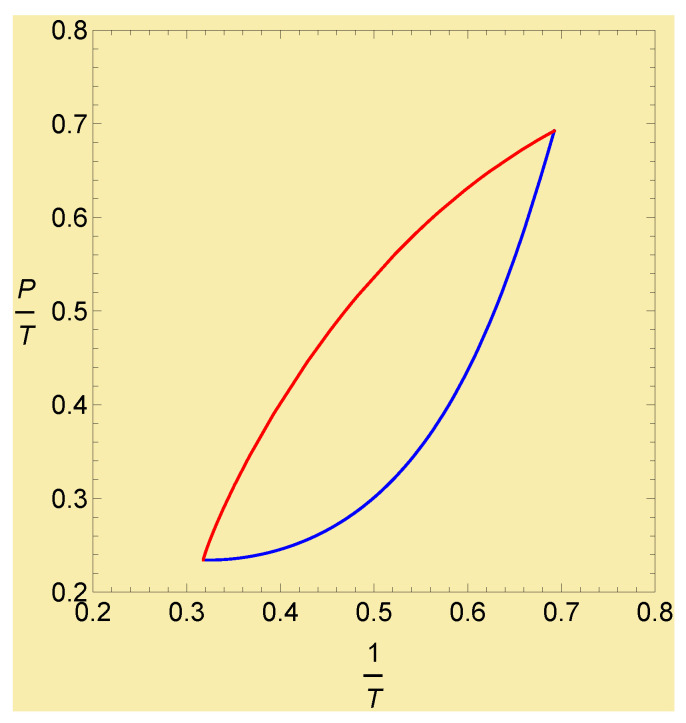
The trajectories (1/T(t), P(t)/T(t)) of the natural gradient flow (blue curve) and *G*-gradient flow (red curve). The parameters and the initial and final values of (u, v) are same as in Figure 1.

We performed a numerical analysis and obtained the numerical solutions. Figure 1 shows the contour plot of the entropic function (60) and the trajectories of the natural- and *G*-gradient flow starting from (u(0),v(0))=(1.5,1.5) to the equilibrium state at $\left(u_{\mathrm{eq}},v_{\mathrm{eq}}\right)=\left(4.5,4.5\right)$. For the same parameters and the initial and final (equilibrium) states, the trajectories on (1/T(t),P(t)/T(t)) of both models are plotted in Figure 2.

## 5. Conclusions

We reconsidered Onsager’s non-equilibrium thermodynamics from the perspective of our previous studies [10,11,12,13] on the gradient flow in IG. As extensions of the OPE, we proposed the two different gradient-flow equations by replacing Onsager’s phenomenological coefficients with the Hessian of −S(X) or with the inverse of the metric *G* in the space spanned by the thermodynamic extensive variables *X*. We call the former the natural gradient-flow model and the latter *G*-gradient-flow model. We considered both gradient-flow models and their relations. In a similar way to how IG works, where the natural gradient-flow equations are nonlinear in the extensive variables $X^{i}$ in general, the dual equations are linear in the intensive variables $a_{i}$. We considered the relationship between both models and showed that the coefficients L(X) in the natural gradient-flow model are the (negative of) the connection coefficient Γ(X,a) in the cotangent bundle. For both models, the flow $dX^{i}/dt$ in tangent space and the flow $da_{i}/dt$ in cotangent space are related by the transport equations as shown in (22) and in (38). We applied both models to the ideal gas and the van der Waals gas models. Since the ideal gas model is too simple, both models for the ideal gas are the same. In contrast, for the van der Waals gas model, both models are different. We performed numerical analysis and obtained the numerical solutions for the natural- and *G*-gradient flow equations. Their trajectories are clearly different as shown in Figure 1 and Figure 2; hence, they describe different thermodynamic processes.

Recently, Bravetti et al. [19] studied asymmetric relaxations within the context of IG. In order to obtain a gradient-flow equation within the context of IG, they used the gradient of the internal energy of a system, whereas in this work, the gradient of the entropic function of a system is used as well as IG. Thus, it should be interesting to extend our method to a gradient flow with respect to an internal energy or free energy.

## Data Availability

The original contributions presented in this study are included in the article. Further inquiries can be directed to the corresponding author.

## Appendix A. Basics of Information Geometry and the Gradient Flow

Historically, IG [9] was developed by considering a parametrized statistical model, which is called an exponential probability distribution function (pdf):

(A1) $$\begin{array}{c} p_{\theta }\left(x\right)=\exp\left[\theta^{i}F_{i}\left(x\right)-\Psi \left(\theta \right)\right]. \end{array}$$

Here, Ψ(θ) is determined from the normalization of $p_{\theta }\left(x\right)$ as $\Psi \left(\theta \right)=\ln\left[\int dx\exp(\theta^{i}F_{i}\left(x\right))\right]$, and its (Legendre) dual potential function $\Psi^{⋆}\left(\eta \right)$ is

(A2) $$\begin{array}{c} \Psi^{⋆}\left(\eta \right)=E_{p_{\theta }}\left[\ln p_{\theta }\left(x\right)\right]=:-S\left(\eta \right), \end{array}$$

where S(η) is the entropic function with respect to $p_{\theta }\left(x\right)$.

However, such an exponential pdf is not necessarily needed if one would like to construct the dually flat structure in IG. For a given convex function Ψ(θ), one can readily construct its dual convex function $\Psi^{⋆}\left(\eta \right)$ and the associated dual affine coordinates $\theta^{i}$ and $\eta_{i}$ as follows.

(A3) $$\begin{array}{c} \theta^{i}=\frac{\partial \Psi^{⋆}\left(\eta \right)}{\partial \eta_{i}},\;\eta_{i}=\frac{\partial \Psi (\theta )}{\partial \theta^{i}},\;\Psi^{⋆}\left(\eta \right)=\theta^{i}\;\eta_{i}-\Psi \left(\theta \right). \end{array}$$

The positive definite matrices $g_{ij}\left(\theta \right)$ and $g^{ij}\left(\eta \right)$ are obtained from the Hessian matrices of the convex function Ψ(θ) and $\Psi^{⋆}\left(\eta \right)$ as

(A4) $$\begin{array}{c} g_{ij}\left(\theta \right)=\frac{\partial \eta_{i}}{\partial \theta^{j}}=\frac{\partial^{2}\Psi \left(\theta \right)}{\partial \theta^{i}\partial \theta^{j}},\;g^{ij}\left(\eta \right)=\frac{\partial \theta^{i}}{\partial \eta_{j}}=\frac{\partial^{2}\Psi^{⋆}\left(\eta \right)}{\partial \eta_{i}\partial \eta_{j}}, \end{array}$$

respectively. These matrices satisfy the relation $g^{ij}\left(\eta \right)\;g_{jk}\left(\theta \right)=\delta_{k}^{i}$, where $\delta_{k}^{i}$ denotes Kronecker’s delta. As is clear from (A4), these matrices are also Jacobian matrices, e.g., $g_{ij}\left(\theta \right)=\partial \eta_{i}\left(\theta \right)/\partial \theta^{j}$. In this way, the matrices $g_{ij}\left(\theta \right)$ and the inverse $g^{ij}\left(\eta \right)$ are simultaneously Jacobi and Hessian matrices, which are distinctive in the dually flat structure of IG.

Since the connection coefficients Γ are not tensors, there exists a coordinate system in which all connection coefficients become zero, and such a coordinate system is called affine. The α-connection $\nabla^{\left(\alpha \right)}$ [9], which is a one-parameter extension ${\left\{\nabla^{\alpha }\right\}}_{\alpha \in R}$ of Levi–Civita’s connection $\nabla^{\left(0\right)}$, and its dual $\nabla^{⋆(\alpha )}$ are defined by their coefficients as

(A5) $$\begin{array}{c} {\Gamma^{\left(\alpha \right)}}_{ijk}\left(\theta \right):=\frac{\left(1-\alpha \right)}{2}\frac{\partial^{3}\Psi \left(\theta \right)}{\partial \theta^{i}\partial \theta^{j}\partial \theta^{k}},\;\Gamma^{⋆(\alpha )\;ijk}\left(\eta \right):=\frac{\left(1+\alpha \right)}{2}\frac{\partial^{3}\Psi^{⋆}\left(\eta \right)}{\partial \eta_{i}\partial \eta_{j}\partial \eta_{k}}, \end{array}$$

respectively. Among the α-connections, α=±1 plays a central role [9]. One readily sees, from (A5), that the connection coefficients ${\Gamma^{\left(1\right)}}_{ijk}\left(\theta \right)$ of $\nabla^{\left(1\right)}$ ($\Gamma^{⋆(-1)\;ijk}\left(\eta \right)$ of $\nabla^{⋆(-1)}$) vanish, and hence, the θ-coordinates (η-coordinates) are affine for the connection $\nabla^{\left(1\right)}$ ($\nabla^{⋆(-1)}$).

A divergence D(p,r) of a set of two states *p* and *r* is a non-negative function providing a measure of how much they differ. We can regard *r* as a reference state. A well-known example of the divergence is relative entropy (or Kullback–Leibler divergence). In IG, the θ- and η-divergence functions are

(A6a) $$\begin{array}{cc} D(\theta ,\theta_{r}) & :=\Psi \left(\theta \right)-\Psi \left(\theta_{r}\right)-\eta_{i}^{r}\left(\theta^{i}-\theta_{r}^{i}\right), \end{array}$$

(A6b) $$\begin{array}{cc} D(\eta ,\eta^{r}) & :=\Psi^{⋆}\left(\eta \right)-\Psi^{⋆}\left(\eta_{r}\right)-\theta_{r}^{i}\left(\eta_{i}-\eta_{i}^{r}\right), \end{array}$$

respectively. Here, the $\theta_{r}$ (or $\eta^{r}$) denotes the θ- (or η-) vector of a reference state. When $\theta =\theta_{r}$, the θ-divergence $D(\theta ,\theta_{r})$ vanishes, and similarly, the η-divergence $D(\eta ,\eta^{r})$ vanishes when $\eta =\eta^{r}$.

Having described the basics of IG, we briefly explain the gradient-flow equations [6,7] in IG. The gradient-flow equations with respect to the θ-divergence function $D(\theta ,\theta_{r})$ with a given fixed $\theta_{r}$ are

(A7) $$\begin{array}{c} \frac{d\theta^{i}}{dt}=g^{ij}\left(\theta \right)\;\frac{\partial D(\theta ,\theta_{r})}{\partial \theta^{j}}=g^{ij}\left(\theta \right)\;\left(\frac{\partial \Psi (\theta )}{\partial \theta^{j}}-\frac{\partial \Psi (\theta )}{\partial \theta^{j}}{|}_{\theta^{j}=\theta_{r}^{j}}\right), \end{array}$$

in the θ-coordinate system. By using the properties (A3) and (A4), the left-hand side of (A7) is rewritten by

(A8) $$\begin{array}{cc} \frac{d\theta^{i}}{dt} & =\frac{\partial \theta^{i}}{\partial \eta_{j}}\frac{d\eta_{j}}{dt}=g^{ij}\left(\theta \right)\frac{d\eta_{j}}{dt}, \end{array}$$

and applying (A3) to the right-hand side of (A7) leads to $g^{ij}\left(\theta \right)\;\left(\eta_{j}-\eta_{j}^{r}\right)$. Consequently, the gradient-flow Equation (A7) in the θ-coordinate system is equivalent to the linear differential equations

(A9) $$\begin{array}{c} \frac{d\eta_{i}\left(t\right)}{dt}=\eta_{i}\left(t\right)-\eta_{i}^{r}, \end{array}$$

in the η-coordinate system. This linearization is one of the merits of the dually flat structure [9] in IG.

The other set (or the dual) of the gradient-flow equations are given by

(A10) $$\begin{array}{c} \frac{d\eta_{i}}{dt}=-g_{ij}\left(\eta \right)\frac{\partial D(\eta ,\eta^{r})}{\partial \eta_{j}}=-g_{ij}\left(\eta \right)\;\left(\frac{\partial \Psi^{⋆}\left(\eta \right)}{\partial \eta_{j}}-\frac{\partial \Psi^{⋆}\left(\eta \right)}{\partial \eta_{j}}{|}_{\eta_{j}=\eta_{j}^{r}}\right), \end{array}$$

in the η-coordinate system. Similarly, they are equivalent to the linear differential equations

(A11) $$\begin{array}{c} \frac{d\theta^{i}\left(t\right)}{dt}=-\theta^{i}\left(t\right)+\theta_{r}^{j}, \end{array}$$

in the θ-coordinate system.

The gradient flow (A7) and (A10) can be related to the Hamilton flow characterized by the following Hamiltonians

(A12a) $$\begin{array}{cc} H(\theta ,\eta ) & =\frac{1}{2}g^{ij}\left(\theta \right)\left(\eta_{i}-\eta_{i}^{r}\right)\left(\eta_{j}-\eta_{j}^{r}\right)-\frac{1}{2}\chi_{\eta }^{2}\left(\theta \right), \end{array}$$

(A12b) $$\begin{array}{cc} H(\eta ,-\theta ) & =\frac{1}{2}g_{ij}\left(\eta \right)\left(\theta^{i}-\theta_{r}^{i}\right)\left(\theta^{j}-\theta_{r}^{j}\right)-\frac{1}{2}\chi_{\theta }^{2}\left(\eta \right), \end{array}$$

respectively. Here, $\chi_{\eta }^{2}\left(\theta \right)$ and $\chi_{\theta }^{2}\left(\eta \right)$ are given by

(A13a) $$\begin{array}{cc} \chi_{\eta }^{2}\left(\theta \right) & :=g^{ij}\left(\theta \right)\left(\frac{\partial \Psi (\theta )}{\partial \theta^{i}}-\frac{\partial \Psi (\theta )}{\partial \theta^{i}}{|}_{\theta^{i}=\theta_{r}^{i}}\right)\left(\frac{\partial \Psi (\theta )}{\partial \theta^{j}}-\frac{\partial \Psi (\theta )}{\partial \theta^{j}}{|}_{\theta^{j}=\theta_{r}^{j}}\right), \end{array}$$

(A13b) $$\begin{array}{cc} \chi_{\theta }^{2}\left(\eta \right) & :=g_{ij}\left(\eta \right)\left(\frac{\partial \Psi^{⋆}\left(\eta \right)}{\partial \eta_{i}}-\frac{\partial \Psi^{⋆}\left(\eta \right)}{\partial \eta_{i}}{|}_{\eta_{i}=\eta_{i}^{r}}\right)\left(\frac{\partial \Psi^{⋆}\left(\eta \right)}{\partial \eta_{j}}-\frac{\partial \Psi^{⋆}\left(\eta \right)}{\partial \eta_{j}}{|}_{\eta_{j}=\eta_{j}^{r}}\right), \end{array}$$

respectively. Note also that H(θ,η) and H(η,−θ) are related through the canonical transformation $\left(\theta^{i},\eta_{i}\right)$ to $\left(\eta_{i},-\theta^{i}\right)$ [12].

It is worth noting that the scalar field $\chi_{\eta }^{2}\left(\theta \right)$ characterizes the rate of the θ-potential, since it is related to the θ-potential function Ψ(θ) as follows.

(A14) $$\begin{array}{cc} \frac{d\Psi (\theta )}{dt} & =\frac{\partial \Psi (\theta )}{\partial \theta^{i}}\frac{d\theta^{i}}{dt}=g^{ij}\left(\theta \right)\frac{\partial \Psi (\theta )}{\partial \theta^{i}}\left(\frac{\partial \Psi (\theta )}{\partial \theta^{j}}-\frac{\partial \Psi (\theta )}{\partial \theta^{j}}{|}_{\theta^{j}=\theta_{r}^{j}}\right)=\chi_{\eta }^{2}\left(\theta \right)-g^{ij}\left(\theta \right)\eta_{i}^{r}\eta_{j}^{r}, \end{array}$$

where the relations (A3) and (A7) are used. Similarly, the scalar field $\chi_{\theta }^{2}\left(\eta \right)$ characterizes the rate of the η-potential as

(A15) $$\begin{array}{c} \frac{d\Psi^{⋆}\left(\eta \right)}{dt}=-\chi_{\theta }^{2}\left(\eta \right)+g_{ij}\left(\eta \right)\theta_{r}^{i}\theta_{r}^{j}. \end{array}$$

Since $-\Psi^{⋆}\left(\eta \right)$ is the entropy S(η) in (A2), the scalar field $\chi_{\theta }^{2}\left(\eta \right)$ characterizes the rate of the entropy dS(η)/dt in the gradient flows.

By using the gradient-flow Equations (A7) and (A10), we see that

(A16) $$\begin{array}{c} \chi_{\eta }^{2}\left(\theta \right)=g_{ij}\left(\theta \right)\frac{d\theta^{i}}{dt}\frac{d\theta^{j}}{dt},\;\chi_{\theta }^{2}\left(\eta \right)=g^{ij}\left(\eta \right)\frac{d\eta_{i}}{dt}\frac{d\eta_{j}}{dt}. \end{array}$$

## Appendix B. Connections to Cotangent Bundles

Here, we review the connections to cotangent bundles, according to Ref. [20]. In order to retain the consistency of the notations in the text, we use *X* for the local coordinates and (X,a) for the canonical coordinates. The local coordinates $X=(X^{i}),i=1,2,\ldots ,n$ on a smooth manifold M generate natural canonical coordinates $\left(X^{i},a_{i}\right)$ on the cotangent bundle $T^{*}M$ and fibered coordinates $\left(X^{i},a_{i},{\dot{X}}^{i},{\dot{a}}_{i}\right)$ on the tangent of the cotangent bundle $TT^{*}M$. A connection Γ on $T^{*}M$ can be represented by three different methods:

(1) By the differential equations
  (A17) $$\begin{array}{c} \frac{da_{i}}{dt}=\Gamma_{ij}\left(X,a\right)\;\frac{dX^{j}}{dt},\;i,j=1,2,\ldots ,n, \end{array}$$
  which are called the transport equations;
(2) By generators $D_{i}$ given by
  (A18) $$\begin{array}{c} D_{i}=\frac{\partial }{\partial X^{i}}+\Gamma_{ij}\left(X,a\right)\;\frac{\partial }{\partial a_{j}}; \end{array}$$
(3) By the characteristic forms
  (A19) $$\begin{array}{c} \theta_{i}=da_{i}-\Gamma_{ij}\left(X,a\right)\;dX^{j}, \end{array}$$

where $\Gamma_{ij}\left(X,a\right)$ are coefficients of the connection Γ(X,a). Be carful not to confuse the connection Γ(X,a) with a connection ∇ on a Riemann manifold. As can be seen from the transport Equation (A17), the connection Γ(X,a) relates $dX^{i}/dt\in TM$ with $da_{i}/dt\in TT^{⋆}M$. We denote the coefficients of a connection ∇ as ${\Gamma^{k}}_{ij}\left(X\right)$, whereas those of Γ(X,a) are $\Gamma_{ij}\left(X,a\right)$. The horizontal lift $\bar{Y}$ of the vector fields *Y* on the base manifold M is defined by

(A20) $$\begin{array}{c} Y=Y^{i}\frac{\partial }{\partial Y^{i}}\;\Rightarrow \;\bar{Y}=Y^{i}D_{i}, \end{array}$$

which states that the horizontal lift $\bar{\partial /\partial Y^{i}}$ of $\partial /\partial Y^{i}$ on M is the generator

(A21) $$\begin{array}{c} D_{i}=\bar{\frac{\partial }{\partial Y^{i}}}. \end{array}$$

The difference

(A22) $$\begin{array}{c} R\left(Y,Z\right):=\left[\bar{Y},\bar{Z}\right]-\bar{\left[Y,Z\right]} \end{array}$$

is a vertical field. Here, [Y,Z] denotes the Lie bracket of the vector fields Y,Z. The bilinear mapping R(·,·) from vector fields to vertical fields is the curvature of the connection. Since $[\partial /\partial Y^{i},\partial /\partial Y^{j}]=0$, we have

(A23) $$\begin{array}{c} \left[D_{i},D_{j}\right]=R\left(\frac{\partial }{\partial Y^{i}},\frac{\partial }{\partial Y^{j}}\right)=R_{ij\ell }\;\frac{\partial }{\partial a_{\ell }}, \end{array}$$

with

(A24) $$\begin{array}{c} R_{ij\ell }:=\Gamma_{ik}\left(X,a\right)\frac{\partial \Gamma_{j\ell }\left(X,a\right)}{\partial a_{k}}-\Gamma_{jk}\left(X,a\right)\frac{\partial \Gamma_{i\ell }\left(X,a\right)}{\partial a_{k}}+\frac{\partial \Gamma_{j\ell }\left(X,a\right)}{\partial X^{i}}-\frac{\partial \Gamma_{i\ell }\left(X,a\right)}{\partial X^{j}}, \end{array}$$

which are the curvature coefficients of the connection Γ(X,a).

A connection Γ is called linear if the parallel transport is linear. In this case, the connection coefficients are linear in *a*, i.e.,

(A25) $$\begin{array}{c} \Gamma_{ij}\left(X,a\right)={\Gamma^{k}}_{ij}\left(X\right)\;a_{k}, \end{array}$$

as well as the curvature coefficients

(A26) $$\begin{array}{c} R_{ij\ell }={R^{k}}_{ij\ell }\;a_{k}, \end{array}$$

where

(A27) $$\begin{array}{c} {R^{k}}_{ij\ell }:=\frac{\partial }{\partial X^{i}}\;{\Gamma^{k}}_{j\ell }\left(X\right)-\frac{\partial }{\partial X^{j}}\;{\Gamma^{k}}_{i\ell }\left(X\right)+{\Gamma^{k}}_{im}\left(X\right){\Gamma^{m}}_{j\ell }\left(X\right)-{\Gamma^{k}}_{jm}\left(X\right){\Gamma^{m}}_{i\ell }\left(X\right). \end{array}$$

It is well-known that when the integrability conditions $\partial a_{j}/\partial X^{i}=\partial a_{i}/\partial X^{j}$, are satisfied, there exists a potential function W(X) such that

(A28) $$\begin{array}{c} a_{i}=\frac{\partial }{\partial X^{i}}W\left(X\right). \end{array}$$

Then, taking the derivative of both sides with respect to the parameter *t*, we have

(A29) $$\begin{array}{c} \frac{da_{i}}{dt}=\frac{\partial^{2}W\left(X\right)}{\partial X^{i}\partial X^{j}}\;\frac{dX^{j}}{dt}. \end{array}$$

Comparing this relation with (A17), it follows that

(A30) $$\begin{array}{c} \Gamma_{ji}\left(X,a\right)=\frac{\partial^{2}W\left(X\right)}{\partial X^{j}\partial X^{i}}=\frac{\partial^{2}W\left(X\right)}{\partial X^{i}\partial X^{j}}=\Gamma_{ij}\left(X,a\right), \end{array}$$

which states that the connection Γ(X,a) is symmetric (or torsion-free). Similarly, we see that

(A31) $$\begin{array}{c} \frac{\partial }{\partial X^{i}}\Gamma_{j\ell }\left(X,a\right)=\frac{\partial^{2}a_{\ell }}{\partial X^{i}\partial X^{j}}=\frac{\partial^{2}a_{\ell }}{\partial X^{j}\partial X^{i}}=\frac{\partial }{\partial X^{j}}\Gamma_{i\ell }\left(X,a\right). \end{array}$$

Next, we introduce the dual potential function $W^{⋆}\left(a\right)$ as the Legendre transform of W(X),

(A32) $$\begin{array}{c} W^{⋆}\left(a\right)=a_{k}\;X^{k}-W\left(X\right). \end{array}$$

We then see that

(A33) $$\begin{array}{c} X^{i}=\frac{\partial W^{⋆}\left(a\right)}{\partial a_{i}}, \end{array}$$

which implies the integrability conditions $\partial^{i}X^{j}=\partial^{j}X^{i}$. Now, since

(A34) $$\begin{array}{c} \frac{\partial X^{n}}{\partial a_{m}}\;\Gamma_{n\ell }\left(X,a\right)=\frac{\partial X^{n}}{\partial a_{m}}\;\frac{\partial a_{\ell }}{\partial X^{n}}=\frac{\partial a_{\ell }}{\partial a_{m}}=\delta_{\ell }^{m}, \end{array}$$

we see that

(A35) $$\begin{array}{c} \Gamma^{mn}\left(X,a\right)=\frac{\partial X^{n}}{\partial a_{m}} \end{array}$$

are the inverse matrix elements of $\Gamma_{mn}\left(X,a\right)$, i.e., $\Gamma^{mn}\left(X,a\right)\;\Gamma_{n\ell }\left(X,a\right)=\delta_{\ell }^{m}$. Taking a partial derivative of both sides, we obtain

(A36) $$\begin{array}{c} \frac{\partial \Gamma^{mn}\left(X,a\right)}{\partial p_{k}}\;\Gamma_{n\ell }\left(X,a\right)=-\Gamma^{mn}\left(X,a\right)\;\frac{\partial \Gamma_{n\ell }\left(X,a\right)}{\partial a_{k}}. \end{array}$$

By multiplying $\Gamma_{jm}\left(X,a\right)$, we have

(A37) $$\begin{array}{c} \frac{\partial \Gamma_{j\ell }\left(X,a\right)}{\partial a_{k}}=-\frac{\partial \Gamma^{mn}\left(X,a\right)}{\partial a_{k}}\;\Gamma_{n\ell }\left(X,a\right)\Gamma_{jm}\left(X,a\right). \end{array}$$

We also see that

(A38) $$\begin{array}{c} \frac{\partial \Gamma^{mn}\left(X,a\right)}{\partial a_{k}}=\frac{\partial^{2}X^{n}}{\partial a_{k}\partial a_{m}}=\frac{\partial^{2}X^{n}}{\partial a_{m}\partial a_{k}}=\frac{\partial \Gamma^{kn}\left(X,a\right)}{\partial a_{m}}. \end{array}$$

Then, from (A37), we have

$$\begin{array}{c} \Gamma_{ik}\left(X,a\right)\frac{\partial \Gamma_{j\ell }\left(X,a\right)}{\partial a_{k}}=-\;\frac{\partial \Gamma^{kn}\left(X,a\right)}{\partial a_{m}}\;\Gamma_{n\ell }\left(X,a\right)\Gamma_{ik}\left(X,a\right)\Gamma_{jm}\left(X,a\right)=\Gamma_{jm}\left(X,a\right)\;\frac{\partial \Gamma_{i\ell }\left(X,a\right)}{\partial a_{m}}. \end{array}$$

Thus, it follows that

(A39) $$\begin{array}{c} R_{ij\ell }:=\Gamma_{ik}\left(X,a\right)\frac{\partial \Gamma_{j\ell }\left(X,a\right)}{\partial a_{k}}-\Gamma_{jk}\left(X,a\right)\frac{\partial \Gamma_{i\ell }\left(X,a\right)}{\partial a_{k}}+\frac{\partial \Gamma_{j\ell }\left(X,a\right)}{\partial X^{i}}-\frac{\partial \Gamma_{i\ell }\left(X,a\right)}{\partial X^{j}}=0. \end{array}$$

Therefore, the system is completely integrable if R=0, i.e., the connection Γ(X,a) is flat.

A smooth function $H\left(X,a\right):T^{⋆}M\to R$, which is constant along all the horizontal curves (i.e., constant on the parallel covectors), is said to be an invariant function of Γ(X,a). This is equivalent to $D_{i}\;H\left(X,a\right)=0$, i.e.,

(A40) $$\begin{array}{c} \frac{\partial H(X,a)}{\partial X^{i}}+\Gamma_{ij}\left(X,a\right)\;\frac{\partial H(X,a)}{\partial a_{j}}=0. \end{array}$$

On a cotangent bundle, a Hamilton function H(X,a) generates a Hamilton vector field $X_{H}$. In canonical coordinates (X,a), the first-order equations of $X_{H}$ are Hamilton’s equations

(A41) $$\begin{array}{c} \frac{dX^{i}}{dt}=\frac{\partial H(X,a)}{\partial a_{i}},\;\frac{da_{i}}{dt}=-\frac{\partial H(X,a)}{\partial X^{i}}. \end{array}$$

The value of the Hamiltonian H(X,a) is a first integral of $X_{H}$, i.e., a constant along the integral curves of $X_{H}$.

Note that by combining (A40) and (A41), we see that

(A42) $$\begin{array}{c} \frac{da_{i}}{dt}=-\frac{\partial H(X,a)}{\partial X^{i}}=\Gamma_{ij}\left(X,a\right)\frac{\partial H(X,a)}{\partial a_{j}}=\Gamma_{ij}\left(X,a\right)\;\frac{dX^{j}}{dt}, \end{array}$$

which is the transport Equation (A17).

## Appendix C. Levi–Civita Connection and Jacobi Matrix

The coefficients ΓkGij of the Levi–Civita connection with respect to a metric *G* are given by

(A43) ΓkGij(X):=12Gkℓ(X)∂Gℓj(X)∂Xi+∂Giℓ(X)∂Xj−∂Gij(X)∂Xℓ.

By using the relations (28) and (34), we have

(A44) ΓkGij(X)=12Gkℓ(X)ηcd∂Jjc(X)Jℓd(X)∂Xi+∂Jic(X)Jℓd(X)∂Xj−∂Jic(X)Jjd(X)∂Xℓ=12Gkℓ(X)ηcd{Jℓd(X)∂Jjc(X)∂Xi+∂Jic(X)∂Xj+Jic(X)∂Jℓd(X)∂Xj−∂Jjd(X)∂Xℓ+Jjc(X)∂Jℓd(X)∂Xi−Jjd(X)∂Jic(X)∂Xℓ}.

For a Jacobi matrix, we see that

(A45) $$\begin{array}{c} \frac{\partial {J_{j}}^{c}\left(X\right)}{\partial X^{i}}=\frac{\partial^{2}\phi^{c}}{\partial X^{i}\partial X^{j}}=\frac{\partial^{2}\phi^{c}}{\partial X^{j}\partial X^{i}}=\frac{\partial {J_{i}}^{c}\left(X\right)}{\partial X^{j}}, \end{array}$$

which is symmetric under changing *i* and *j*. Then, we see that

(A46) $$\begin{array}{c} {J_{i}}^{c}\left(X\right)\left(\frac{\partial {J_{\ell }}^{d}\left(X\right)}{\partial X^{j}}-\frac{\partial {J_{j}}^{d}\left(X\right)}{\partial X^{\ell }}\right)=0,\;\eta_{cd}{J_{j}}^{c}\left(X\right)\frac{\partial {J_{\ell }}^{d}\left(X\right)}{\partial X^{i}}-\eta_{cd}{J_{j}}^{d}\left(X\right)\frac{\partial {J_{i}}^{c}\left(X\right)}{\partial X^{\ell }}=0, \end{array}$$

because $\eta_{cd}$ is diagonal. Consequently, it follows that

(A47) ΓkGij(X)=Gkℓ(X)︸ηabJka(X)JℓbηcdJℓd(X)∂Jjc(X)∂Xi=ηabηcd︸δcaJka(X)Jℓb(X)Jℓd(X)︸δbd∂Jjc(X)∂Xi=Jka(X)∂Jja(X)∂Xi.
